# Dietary Patterns and Age-Related Macular Degeneration: A Matched Case–Control Study

**DOI:** 10.3390/nu18101582

**Published:** 2026-05-15

**Authors:** Mougni Mohamed Azalati, Hong Jiang, Kejing Zhang, Liyun Kong, Lina Wang, Zhaofang Li, Yahui Fan, Fangyao Chen, Le Ma, Wei Zhang

**Affiliations:** 1 School of Public Health, Xi’an Jiaotong University Health Science Center, Xi’an 710061, China; mouigniaza91@gmail.com (M.M.A.); jiangh0926@outlook.com (H.J.); zhangkejing2022@163.com (K.Z.); kongly@xjtu.edu.cn (L.K.); wln305@stu.xjtu.edu.cn (L.W.); 13821175362@163.com (Z.L.); fyh14042166@stu.xjtu.edu.cn (Y.F.); chenfy@xjtu.edu.cn (F.C.); 2 Key Laboratory of Environment and Genes Related to Diseases (Xi’an Jiaotong University), Ministry of Education of China, Xi’an 710061, China

**Keywords:** dietary pattern, eating behavior, age-related macular degeneration, principal component analysis

## Abstract

**Background**: Previous research on diet and age-related macular degeneration (AMD) has emphasized primarily particular nutrients or foods, and the influence of comprehensive dietary patterns that represent actual eating behaviors is largely unknown. **Objective**: The aim of this study was to assess the association between dietary patterns and the odds of AMD. **Methods**: A case–control study involving 246 participants with AMD and 246 controls are individually matched by age and gender. Dietary patterns were identified through principal component analysis using a validated food frequency questionnaire. Multivariable conditional logistic regression models were applied to examine the association between the extracted dietary patterns and the likelihood of AMD. **Results**: Three major dietary patterns were found, accounting for 50.59% of the total variance explained. The prudent dietary pattern represented a high intake of vegetables, fruits, soybeans and its products, edible fungi and algae, and nuts were associated with reduced odds ratios (ORs) of the highest tertile compared to the lowest tertile (OR, 0.29, 95% confidence interval [CI], 0.14−0.59, *p* for trend = 0.001). The estimated likelihood for AMD in the highest tertile of egg and milk dietary pattern intake, which is characterized by a high intake of eggs, milk and dairy products, and refined grains, was 0.40 (95% CI, 0.23−0.67, *p* for trend < 0.001) compared with those in the lowest tertile. No association with AMD was identified for the animal dietary pattern (*p* > 0.05). **Conclusions**: Adherence to dietary patterns rich in fruits, vegetables, nuts, refined grains, eggs, milk and dairy products is associated with reduced odds of AMD, emphasizing the potential relevance of dietary habits to visual health among middle-aged and elderly adults.

## 1. Introduction

Age-related macular degeneration (AMD) is the leading cause of vision impairment in the developed world for persons over 50 years [1]. The global prevalence of AMD is anticipated to grow significantly, and the number of affected persons is expected to increase from approximately 196 million in 2020 to 288 million by 2040 [2]. Existing therapies have shown the ability to inhibit the progression of certain types of AMD, but are intrusive and unable to reverse existing retinal damage [3]. Therefore, it is essential to determine and develop feasible AMD prevention strategies.

Evidence from in vitro and animal experiments demonstrated that oxidative damage and inflammation are key contributors in the pathogenesis of AMD [4,5]. A diet rich in antioxidant properties has been identified to play a beneficial function for the retina by alleviating oxidative stress and inflammation [6,7]. Over recent decades, numerous studies have examined the association between diet with the development of AMD [8,9,10]. However, previous investigations were mainly concentrated on specific nutrients, foods, or food groups and have yielded mixed results [11,12,13]. The discrepancies in these findings might be that people do not eat isolated nutrients, and their typical diet consists of various foods with complex dietary components that may interact or synergize. In this context, dietary pattern analysis assesses overall dietary consumption, considering the complexity, quality, variation, and food interactions, giving an alternative method for assessing nutritional influences on AMD risk [14,15,16]. Principal component analysis (PCA) is a common data-driven approach that identifies foods frequently consumed in combination and integrates food items or groups based on their correlation, which has been applied to discern dietary patterns associated with health outcomes and its risk factors in the prior literature [17,18,19]. To date, limited research has assessed the association between dietary patterns with the odds of AMD, predominantly conducted among Caucasian populations [14,15,20]. This lack of ethnic diversity in research populations raises essential questions about the generalizability of current dietary recommendations for AMD prevention across different racial and ethnic groups.

Therefore, in the current investigation, an analysis was conducted to examine the association between dietary patterns identified through PCA with the likelihood of AMD in a Chinese population, which would provide evidence for healthy lifestyle approaches in promoting the health of the elderly.

## 2. Materials and Methods

### 2.1. Study Population and Data Collection

The study population was drawn out of Xi’an Eye Study data. The study design details have been reported elsewhere [21]. In brief, eligible individuals were recruited by posting advertisements and flyers from clinics and health fairs in Xi’an, Shaanxi Province, China. Each participant underwent a comprehensive ophthalmic examination by experienced ophthalmologists using a standard protocol, including the best-corrected visual acuity, intraocular pressure, slit lamp inspection, optical coherence tomography, fundus photography, and fundus autofluorescence. All participants underwent a questionnaire on personal demographics, lifestyle characteristics, dietary intake, medical history, and other health-related information.

The current study had a case–control design. Potential AMD cases were aged ≥45 years and clinically diagnosed with early AMD (soft drusen and/or pigmentary abnormalities) or late AMD (geographic atrophy or signs of exudative AMD) by eye specialists according to the Age-Related Eye Disease Study (AREDS) classification system [22]. Cases with high myopic patients, glaucoma, clinically severe diabetic retinopathy, and those with a history of any ocular condition that might possibly affect central or parafoveal macular visual function were excluded. Potential patients who had undergone intraocular surgery within the previous six months, who were taking photosensitizing medicines (such as phenothiazines and chloroquine) that might influence visual function, who followed special diets (such as vegetarians and patients with cancer or Crohn’s disease) were also not allowed to participate. Participants who left 10 or more items blank on the food frequency questionnaire (FFQ) or reported implausible daily caloric intake (outside the range of 800–4200 kcal/d for men and 500–3500 kcal/d for women) were also excluded.

Controls were selected using the same inclusion and exclusion criteria as cases and were free from AMD. Following exclusion, cases and controls were matched by age and gender; finally, an overall number of 246 cases and 246 controls were incorporated in the current analysis.

The study was conducted in compliance with the guidelines in the Declaration of Helsinki and received ethical approval from the Ethics Committee of Xi’an Jiaotong University (2014-154). Prior to enrollment, the research aims, procedures, and potential risks and benefits were fully explained to each participant, and all of the participants provided written informed consent before participation.

### 2.2. Dietary Patterns Assessment

The dietary information was obtained through a semi-quantitative validated FFQ comprising 59 food items divided into 11 food groups: vegetables, fruits, edible fungi and algae, soybeans and its products, refined grains, nuts, red meat, poultry meat, fish and seafood, eggs, milk and dairy products (Supplementary Table S1). Participants reported their average consumption frequency over the preceding year, with five categorical responses ranging from; per day, per week, per month, per year, or never. Standard portion sizes were carefully defined based on typical consumption patterns. For the majority of food items (e.g., vegetables, red meat, and staple foods), the reference option was standardized to 100 g per serving. Natural units or standard household measures were applied where more biologically or culturally appropriate (e.g., one medium egg, one glass of milk, or one standard bowl). To enhance the precision of portion size estimation and minimize recall bias, a standardized color food photography atlas was utilized during face-to-face assessments, providing participants with intuitive visual representations of various portion sizes for the surveyed food items. Daily energy and nutrient intake were calculated by multiplying the equivalent nutrient contribution of a standard portion (100 g) for each food item and summing across all items. The FFQ showed a reasonable validity in a subsample validation study, with Spearman correlation coefficients against three-day, 24-h dietary recalls generally ranging from 0.40 to 0.80 for most food items. Reproducibility was further confirmed by intraclass correlation coefficients ranging from 0.60 to 0.94 across major food items over a one-year interval, indicating adequate reliability of the dietary assessments.

### 2.3. Dietary Patterns Identification

The current study employed PCA to extract dietary patterns from food groups, before applying PCA, the suitability of data for PCA was evaluated using the Kaiser–Meyer–Olkin (KMO) measure and Bartlett’s test of sphericity (BTS) [23,24]. The number of dietary patterns was determined by eigenvalues (>1.0), scree plot inspection, Horn’s parallel analysis (incorporating 1000 Monte Carlo simulations), cumulative variance explained, and nutritional interpretability. Orthogonal rotation (varimax) has been employed to maximize the cumulative variances of each component loading as well as to enhance the understanding regarding the identified dietary patterns. To assess the structural stability of the extracted dietary patterns, sensitivity analyses were conducted using two alternative oblique rotational approaches (Direct Oblimin and Promax). Food groups with absolute factor loadings >0.30 were regarded as significant contributors, and patterns were labeled based on these dominant components [25]. Factor scores were determined by adding the products of food intake with their corresponding loadings; a greater score indicates more adherence to that specific pattern [26]. To evaluate the internal reproducibility and stability of the observed dietary patterns, a non-parametric bootstrap resampling procedure with 2500 replications was conducted. Variable-level stability was defined as the proportion of bootstrap samples in which each food group retained a factor loading ≥0.30 on the same component. Additionally, Tucker’s congruence coefficient was calculated to quantify the overall structural similarity between the original loading vectors and the bootstrapped samples.

### 2.4. Assessment of Covariates

Information on demographics, lifestyle, medical history, and other health-related factors, including age, gender, educational attainment, smoking status, alcohol consumption, sun leakage and protection, physical activity, supplement use, family history of AMD, and history of coronary heart disease, was collected through a structured questionnaire. Physical activity was calculated by multiplying energy expenditure in metabolic equivalent tasks (METs) per hour per week by the duration of each activity. The cumulative values of all activities were then summed to produce the overall physical activity levels. Participants’ levels of physical activity were classified into three categories: low (<8.3 METs-h/w), moderate (8.3–16.7 METs-h/w), and high (>16.7 METs-h/w) [27]. Body mass index (BMI) was determined as weight in kilograms divided by the square of height in meters [28].

### 2.5. Statistical Analysis

The required sample size in this matched case–control study was determined using an estimated odds ratio (OR) of 2.0, 90% power, and a significance threshold of 0.05. The corresponding critical values were $Z_{1-\alpha /2}$ = 1.96 and $Z_{\beta }$ = 1.28. The formula used for matched pairs is as follows:

$$n=\frac{{\left[Z_{1-\alpha /2}/2+Z_{\beta }\sqrt{P\left(1-P\right)}\right]}^{2}/{\left(P-0.5\right)}^{2}}{P_{0}\left(1-P_{1}\right)+P_{1}\left(1-P_{0}\right)}$$

where n represents the total number of pairs required for the study. $Z_{1-\alpha /2}\;$ and $Z_{\beta }\;$ denote the critical values from the standard normal distribution that correspond to the significant level *α* and statistical power *β*, respectively. $P_{0}\;$ and $P_{1}\;$ indicate the estimated exposure rates proportion for the specific factor in the control and case groups, respectively. This yielded approximately 246 matched pairs of cases and controls, and an extra 10–20% was added to account for potential dropouts or missing data. To evaluate the adequacy of the sample size relative to the number of predictors, the events per variable (EPV) was calculated. With 246 cases and 10 predictors in the multivariable models, the resulting EPV was 24.6, which exceeds the recommended threshold of EPV ≥ 10, minimizing the risk of overfitting and ensuring the stability of the coefficient estimates.

Characteristics of participants according to cases and controls for categorical variables were reported as numbers (percentages) and as mean (standard deviation) or median (P25, P75) for continuous variables. Group differences were examined using the chi-square test for categorical variables and ANOVA or the Kruskal–Wallis test for continuous variables.

Individuals were classified into tertiles according to their scores for each dietary pattern. Potential covariates were identified through a comprehensive literature review of established AMD risk factors and further screened using univariate analysis (Supplementary Table S2). Factors demonstrating a significant or marginal association with AMD (*p* < 0.1) were considered eligible for inclusion in the multivariable models. Conditional logistic regression models have been used to determine the ORs and 95% confidence intervals (CIs) for the associations of each dietary pattern and the likelihood of AMD, using the lowest tertile as the reference. The crude model estimate has been obtained by adjusting for matching factors. Multivariate model was adjusted for educational attainment (less than college or college and above), smoking status (never, past, or current), alcohol consumption (yes or no), physical activity (low, moderate, or high), and BMI (continuous). The fully adjusted model was further adjusted for supplement use (yes or no), family history of AMD (yes or no), history of coronary heart disease (yes or no), and total energy intake (continuous). Trends across categories were analyzed by designating the median value of each group as a continuous variable in the multivariate models. In addition, restricted cubic spline regressions with three knots at the 10th, 50th, and 90th percentiles were applied to examine potential dose−response associations between dietary patterns with the likelihood of AMD. E-values were calculated to evaluate the potential impact of unmeasured confounding on the observed associations. Higher values indicate that stronger unmeasured confounding would be required to attenuate the results to the null. To explore potential effect modification, the analysis was further stratified by BMI (<24 vs. ≥24 kg/m^2^), physical activity (<median vs. ≥median), smoking status (yes vs. no), and alcohol consumption (yes vs. no). The significance of interactions was evaluated by contrasting models with and without the interaction terms using likelihood ratio tests. Several sensitivity analyses were implemented to evaluate the robustness of the findings of the current study by adjusting for the number of hours of sunlight exposure and sun protection as a confounder; adjusting for dietary intake of lutein–zeaxanthin; evaluating results within a non-matched population; and excluding participants with prevalent type 2 diabetes, hypertension, or coronary heart disease. Ordinal logistic regression was also performed to investigate the associations between these dietary scores and the odds of AMD. To assess the risk of overfitting and enhance model generalizability, a least absolute shrinkage and selection operator (LASSO) logistic regression with 10-fold cross-validation was conducted. The optimal regularization parameter (λ) was determined based on the minimum cross-validation mean deviance.

Model adequacy was verified using the Pregibon link test and Information Criteria, with multicollinearity evaluated via variance inflation factors (VIFs). No multicollinearity was observed, as all VIFs were <1.20.

All the statistical analyses were performed using Stata version 16.0 (Stata Corp., College Station, TX, USA), and statistical significance was set at *p* < 0.05.

## 3. Results

### 3.1. Dietary Pattern

Three major dietary patterns were identified, collectively explaining a moderate 50.59% of the total variance in dietary intake (Table 1 and Supplementary Figure S1). Parallel analysis confirmed retention of three components, which was fully consistent with the inflection point observed in the scree plot (Supplementary Table S3). The suitability test for factor analysis indicated a KMO value of 0.76, and Bartlett’s sphericity was statistically significant *p* < 0.001, supporting the appropriateness of PCA. The factor structures derived from oblique rotation methods were identical with the original orthogonal Varimax rotation (Supplementary Tables S4 and S5), further confirming the stability of the identified patterns. Bootstrap analysis confirmed the stability of the identified patterns. High−loading food groups consistently suggested robust factor structures across the 2500 replications, with most core variables retaining identical loading in ≥80% of the bootstrap samples (Supplementary Table S6).

The component that labeled the prudent dietary pattern was characterized by high intakes of vegetables, fruits, soybeans and its products, edible fungi and algae, and nuts; the egg and milk dietary pattern was characterized by high intakes of eggs, milk and dairy products, and refined grains; whereas high intakes of poultry, fish and seafood, and red meat characterized an animal foods dietary pattern.

### 3.2. Characteristics of the Study Population According to the Tertiles of Dietary Pattern

Participants with greater scores on the prudent pattern were slightly more likely to be men, more likely to be users of supplements, and more likely to have a history of coronary heart disease compared to those with lower scores for this pattern. Participants who adhered more closely to the egg and milk dietary pattern tended to be less likely to have a family history of AMD or coronary heart disease and highly likely to be more educated than those with less adherence to this pattern. For the animal foods dietary pattern, participants who scored higher were mostly men, more prone to smoking, had a higher prevalence of reported family history of AMD, and had less intake of supplements than those with lower scores on this pattern (Table 2).

### 3.3. Association Between Dietary Patterns and the Likelihood of AMD

**Figure 1:**
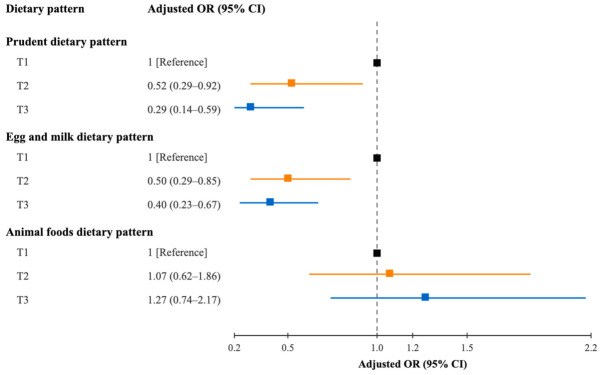
Forest plot summarizing multivariate logistic regression models for the association between dietary patterns with the odds of AMD. The logistic models were adjusted for educational attainment, smoking status, alcohol consumption, physical activity, BMI, supplement use, family history of AMD, history of coronary heart disease, and total energy intake. Each square represents the OR point estimate for the corresponding tertile, with horizontal lines indicating the 95% CIs. The black square represents the reference tertile 1, the orange line and blue line represent tertile 2 and 3. AMD: age-related macular degeneration; CI: confidence interval; OR: odds ratio.

**Figure 2:**
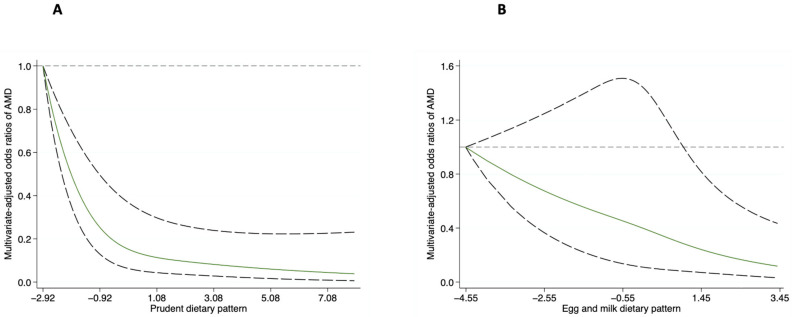
Restricted cubic spline analysis of the association between a prudent dietary pattern (**A**) and egg and milk dietary pattern (**B**) with the odds of AMD. The logistic models were adjusted for educational attainment, smoking status, alcohol consumption, physical activity, BMI, supplement use, family history of AMD, history of coronary heart disease, and total energy intake. The solid green line represents the odds ratio, and the dashed line represents the 95% confidence interval. AMD: age-related macular degeneration.

Higher intake of a prudent dietary pattern was associated with a reduced likelihood of AMD in the crude model. The magnitude of the association did not materially alter after adjustment for demographic and lifestyle variables. In the fully adjusted model, the observed association was slightly attenuated but remained significant; participants with the highest score presented a reduced odds of AMD (OR = 0.29; 95% CI: 0.14–0.59; *p* = 0.001). A similar inverse association was observed for the egg and milk dietary pattern. When comparing the top to the bottom tertiles, the corresponding multivariate adjusted OR was 0.40 (95% CI: 0.23–0.67) (Figure 1). Findings from restricted cubic spline regressions revealed a linear association for both the prudent dietary pattern and egg and milk dietary pattern with the likelihood of AMD (both *p*_linearity_ < 0.001). The ORs for AMD were 0.64 (95% CI: 0.48–0.87) and 0.56 (95% CI: 0.34–0.93) per 1 SD increase in prudent and egg and milk scores, respectively (Figure 2). The animal foods dietary pattern failed to show a significant association with the odds of AMD (Supplementary Table S7). The association of dietary pattern and the likelihood of AMD was similar across strata of BMI, physical activity, smoking status, and alcohol consumption. No significant interactions were detected between the dietary pattern and these potential AMD risk factors (Supplementary Table S8).

### 3.4. Sensitivity Analysis

In sensitivity analyses, the significant associations remained unchanged after adjusting for the number of hours of sunlight exposure and sun protection, comparing the extreme to the lowest tertile of prudent, egg and milk, and animal foods dietary patterns with ORs of 0.24 (95% CI: 0.11–0.49), 0.40 (95% CI: 0.22−0.61), and 1.30 (95% CI: 0.78−2.16), respectively. Similarly, after additionally adjusting for dietary intake of lutein–zeaxanthin, the observed associations remained materially unchanged. After restricting the analyses to results within a non-matched population, the sensitivity analyses did not materially alter the associations. Furthermore, the associations with AMD remained largely similar to the results from primary analyses after excluding participants who had type 2 diabetes, hypertension, or coronary heart disease (Supplementary Table S9). For the prudent dietary pattern, the E-value for the point estimate was 6.36 (lower bound: 2.77); similarly, for the egg and milk dietary patterns, the E-value was 4.44 (lower bound: 2.34), collectively indicating that a substantial degree of unmeasured confounding would be necessary to fully attenuate the observed associations to the null (Supplementary Table S10). The optimal regularization parameter was identified as λ = 0.006, yielding a minimum cross-validation mean deviance of 1.37, with 10 predictors retained at non-zero coefficients. The consistency between LASSO-derived predictors and those in the primary model indicated no evidence of overfitting.

## 4. Discussion

Three major dietary patterns were found in the present study; greater adherence to the prudent dietary pattern, characterized by a high intake of vegetables, fruits, soybean and its products, edible fungi and algae, and nuts, was associated with reduced AMD odds in a dose–response manner. The egg and milk dietary pattern, reflecting a high consumption of eggs, milk and dairy products, and refined grains, was similarly associated with a lower AMD likelihood. These results provide further evidence that the prudent and egg and milk dietary patterns were inversely associated with the odds of AMD.

Several observational studies have demonstrated that a specific diet might be associated with reduced odds of AMD. However, most of these investigations were mainly concentrated on a single food or nutrient, which might ignore the intricate interplay between foods and nutrients [29,30,31]. In contrast, dietary pattern analysis provides a comprehensive framework for evaluating dietary influences by considering several food categories rather than specific foods or nutrients. This approach can reveal synergistic interactions among dietary components and provides contextual insight into overall eating habits, and it has been widely utilized to evaluate connections between food and chronic diseases and to help develop evidence-based nutritional recommendations [32,33]. In this context, dietary pattern analysis was increasingly employed to examine the association between overall diet and the likelihood of AMD. The results of the current study are in line with previous findings on healthy dietary patterns. Nevertheless, the magnitude of the observed associations in the present study appears to be slightly stronger than those reported in previous studies conducted in Western populations. This discrepancy may be attributed to the specific food components within the prudent dietary pattern. For instance, soybeans and edible fungi contain isoflavones and specific carotenoids that have been previously inversely linked to retinal oxidative stress [34]. Additionally, traditional Chinese stir-frying, which combines thermal processing with an oil matrix, could hypothetically enhance the bioavailability of fat-soluble nutrients like carotenoids compared to raw consumption [35,36]. Nevertheless, although these biological mechanisms provide a plausible explanation for the present results, they may not fully account for the magnitude of the reported risk estimates, and the results should be interpreted with caution and warrant confirmation in future studies with a larger population.

Although the mechanism behind the observed associations remains unclear, biologically plausible pathways have been proposed. The retina’s exceptionally high metabolic activity renders it susceptible to oxidative damage, which might be attenuated by the antioxidants characteristics of the prudent dietary pattern [37,38]. Furthermore, the phospholipid-rich composition of the ‘Egg and Milk’ dietary pattern could theoretically facilitate the delivery of these antioxidant to retinal tissues via mixed micelle formation [39,40]. However, given the observational nature of this case–control study, these mechanistic pathways remain highly speculative, and further research is warranted to better understand the underlying pathways.

Several limitations warrant consideration when interpreting the present findings. First, due to the observational nature of the study, the possibility of reverse causality cannot be excluded, as individuals diagnosed with AMD may have altered their dietary habits. However, given that early-stage AMD often progresses without noticeable symptoms, participants maintaining high visual acuity may be less motivated to alter their habitual diet or lifestyle [41]. Second, the dietary information used to derive dietary patterns was obtained through FFQ, which comes with inherent limitations. Although the FFQs utilized in the current study were validated against a dietary record, some degree of exposure misclassification and recall bias were inevitable. In particular, the standardized food list may not have captured all culturally specific variations or rarely consumed food items, potentially failing to account for the full variability of participants’ actual habitual intake. In addition, reliance on long-term memory and challenges in estimating portion sizes may have led to misreporting and could have influenced the accuracy of the results. Moreover, self-reported data are susceptible to social desirability bias, where individuals may tend to overreport the consumption of healthy foods and underreport unhealthy items to align with social expectations. Furthermore, the possibility of differential misclassification between cases and controls must be acknowledged. Given the retrospective self-reported nature of the FFQ, individuals diagnosed with AMD may exhibit different recall patterns compared to healthy controls, potentially overestimating or altering the reporting of their dietary habits in response to their diagnosis. While our quantitative probabilistic bias analysis suggested that such differential misclassification would likely attenuate rather than overestimate the observed associations (Supplementary Table S11), it cannot be entirely eliminated and may still influence the precision of the estimated relationships. In addition, challenges in quantifying precise portion sizes may also contribute to misclassification bias. Third, despite the observed associations remaining largely consistent after adjustment for several potential confounders, the possibility of residual confounding cannot be fully eliminated. While educational attainment was included as a proxy for socioeconomic status, it may not fully capture the broader socioeconomic disparities that influence dietary behavior and health consciousness, leaving the possibility that residual socioeconomic confounding partially contributes to the observed associations [42]. In addition, outdoor sunlight exposure represents another potential source of confounding, as ultraviolet (UV) radiation has been shown to induce oxidative stress and cumulative photochemical damage to the macula [43]. Although self-reported daily sunlight exposure hours and sun protection use were incorporated as covariates in sensitivity analyses to mitigate this influence, the inherent limitations of self-reported measures mean that residual confounding from UV exposure cannot be entirely excluded. Furthermore, elevated circulating concentrations of lutein/zeaxanthin have been consistently shown to have a strong inverse association with the risk of AMD, raising the possibility that these circulating biomarkers could confound the observed associations between dietary patterns and AMD. Despite a reasonable correlation observed between self-reported lutein/zeaxanthin intake and plasma biomarker levels (r = 0.13, *p* = 0.03), residual confounding attributable to recall bias, measurement error, and dietary misclassification remains inevitable. Moreover, due to the lack of genetic variant data (e.g., CFH and ARMS2) in the current study, we were unable to account for the potential confounding effects of genetic susceptibility. Although the E-value analyses suggest that the identified associations are robust against unmeasured confounding, the possibility that residual confounding partially explains these estimates cannot be completely ruled out. Future studies integrating genetic and serological data are warranted to further clarify and validate these findings. Fourth, although bootstrap resampling was used to demonstrate the internal stability of the dietary patterns, the absence of validation in an independent population remains a limitation. Furthermore, the derived patterns explained a moderate proportion (50.59%) of the total variance, capturing the primary dietary structures in this population. However, the remaining unexplained variance indicates that PCA-derived patterns may not fully encompass the inherent complexity of individual dietary behaviors. Further research is required to determine whether alternative patterning solutions or additional dietary components could better explain the associations with AMD. Finally, recruitment through advertisements and health promotion events may preferentially attract individuals who are more health-conscious, socially active, or have better access to healthcare. Furthermore, given the substantial regional variations in culinary traditions and dietary habits, the dietary patterns observed in the current study may be specific for the local culture. Therefore, the results may not be directly generalizable to the broader population or other ethnic groups with distinct food practices. Future multi-center studies involving more diverse populations are warranted to verify and extend these findings.

## 5. Conclusions

In summary, the results of the current study demonstrated that a diet that was abundant in fruits, vegetables, eggs, milk and dairy products, nuts, and refined grains was associated with reduced odds of AMD. These findings provide additional epidemiological evidence regarding associations between specific dietary patterns and AMD, suggesting that higher adherence to a dietary pattern, characterized by antioxidants and vitamins, exhibited lower odds of AMD. Nevertheless, further investigative efforts are required to confirm these associations and clarify the underlying biological mechanism.

## Figures and Tables

**Table 1:** Identification of dietary patterns *.

Variables	Prudent Dietary Pattern	Egg and Milk Dietary Pattern	Animal Foods Dietary Pattern
Vegetables	0.44	−	−
Soybeans and its products	0.52	−	−
Edible fungi and algae	0.44	−	−
Fruits	0.41	−	−
Fish and seafood	−	−	0.62
Nuts	0.34	−	−
Refined grains	−	0.32	−
Eggs	−	0.53	−
Milk and dairy products	−	0.50	−
Red meat	−	−	0.38
Poultry	−	−	0.60
Total initial eigenvalues % of variance explained	20.53	15.54	14.52
Cumulative %	20.53	36.07	50.59

* Numbers represent the factor loading for each food category or food item, and the components were identified by items with an absolute value greater than 0.30. Variance explained and cumulative are represented as percentages.

**Table 2:** Characteristics of the study participants according to the tertiles of the dietary pattern scores.

Variables	Prudent Dietary Pattern		*p*-Value	Egg and Milk Dietary Pattern		*p*-Value	Animal Foods Dietary Pattern		*p*-Value
	T1	T3		T1	T3		T1	T3	
Age, y	63.94 ± 7.12	65.91 ± 7.29	0.04	65.15 ± 7.22	64.80 ± 7.91	0.75	65.77 ± 7.12	64.20 ± 7.56	0.11
Male	85 (51.52)	59 (36.20)	0.01	66 (40.00)	81 (49.69)	0.17	68 (40.96)	88 (53.66)	0.04
Educational attainment			0.51			0.03			0.24
Less than college	83 (50.30)	72 (44.17)		73 (44.24)	70 (42.94)		86 (51.81)	70 (42.68)	
College and above	82 (49.70)	91 (55.83)		92 (55.76)	93 (57.06)		80 (48.19)	94 (57.32)	
Current smoker	35 (21.21)	23 (14.11)	0.12	26 (15.76)	33 (20.25)	0.34	39 (17.47)	43 (26.22)	0.02
Alcohol consumption	20 (12.12)	18 (11.04)	0.50	19 (11.52)	18 (11.04)	0.36	20 (12.05)	25 (1524)	0.50
Physical activity			0.12			0.06			
Low	134 (81.21)	115 (70.55)		126 (76.36)	108 (66.26)		116 (69.88)	123 (76.83)	0.32
Moderate	20 (12.12)	32 (19.63)		23 (13.94)	34 (20.86		30 (18.07)	28 (17.07)	
High	11 (6.67)	16 (9.82)		16 (9.70)	21 (12.88)		20 (12.05)	10 (6.10)	
Sun leakage and protection	39 (23.64)	42 (25.77)	0.88	44 (26.67)	48 (29.45)	0.07	41 (24.70)	43 (26.22)	0.89
Body mass index, kg/m^2^	23.39 ± 3.32	23.44 ± 2.79	0.92	23.33 ± 3.19	23.40 ± 2.92	0.93	23.52 ± 3.00	23.56 ± 3.25	0.25
Supplement use	51 (30.91)	79 (48.47)	0.001	73 (44.24)	75 (46.01)	0.19	88 (53.01)	55 (33.54)	>0.99
Family history of AMD	23 (14.02)	32 (19.63)	0.32	38 (23.17)	25 (15.34)	0.004	22 (13.25)	40 (24.39)	>0.99
History of coronary heart disease	27 (16.36)	46 (28.22)	0.04	52 (31.52)	33 (20.25)	>0.99	37 (22.29)	38 (23.17)	0.89
Hypertension	53 (32.12)	55 (33.74)	0.87	50 (30.30)	55 (33.74)	0.48	61 (36.75)	51 (31.10)	0.53
Type 2 diabetes	28 (16.97)	21 (12.88)	0.56	28 (16.97)	25 (15.34)	0.66	30 (18.07)	22 (13.41)	0.45
Total energy intake, kcal/d	1634.30 (1252.31, 1913.66)	2878.86 (2330.87, 3350.37)	<0.001	2146.61 (1423.54, 2769.27)	2377.14 (1832.87, 2811.52)	0.01	2572.38 (1943.30, 3054.99)	2155.08 (1524.54, 2621.76)	<0.001

AMD, age-related macular degeneration; data are expressed as mean ± standard deviation or median (P25, P75) for continuous variables or numbers (percentages) for categorical variables. Discrepancies in dietary pattern scores across groups were tested using ANOVA, Kruskal–Wallis, or chi-square test as appropriate.

## Data Availability

The original contributions presented in this study are included in the article/Supplementary Material. Further inquiries can be directed to the corresponding authors.

## Supplementary Materials

The following supporting information can be downloaded at: https://www.mdpi.com/article/10.3390/nu18101582/s1. Figure S1: Scree plot of dietary components of subjects. The abscissa is the component number, and the ordinate is the eigenvalue of the dietary components. Table S1: Food groups and items used in the principal component analysis. Table S2: Characteristics of participants according to age-related macular degeneration status. Table S3: Parallel analysis of eigenvalues and retained components in PCA. Table S4: Dietary pattern factor loading using Oblimin rotation. Table S5: Dietary pattern factor loading using Promax rotation. Table S6: Stability evaluation analysis of factor loading for the identified dietary patterns across 2500 bootstrap replications. Table S7: Odds ratios (95% CIs) for age-related macular degeneration across tertiles of dietary pattern scores. Table S8: Stratified analyses of dietary patterns and the likelihood of AMD. Table S9: Sensitivity analyses of the associations between dietary components and age-related macular degeneration. Table S10: Robustness to unmeasured confounding using E-values. Table S11: Probabilistic bias simulation results adjusting for differential misclassification.

## Abbreviations

The following abbreviations are used in this manuscript:

AMD	Age-related macular degeneration
AREDS	Age-Related Eye Disease Study
BMI	Body mass index
BTS	Bartlett’s test of sphericity
CI	Confidence interval
FFQ	Food frequency questionnaire
KMO	Kaiser–Meyer–Olkin
LASSO	Least absolute shrinkage and selection operator
METs	Metabolic equivalent tasks
OR	Odds ratio
PCA	Principal component analysis
UV	Ultraviolet
