# Supplementary material for: Dietary Patterns and Age-Related Macular Degeneration: A Matched Case–Control Study
Source: Nutrients. 2026 May 15;18(10):1582. doi: 10.3390/nu18101582 (PMC13209175; doi:10.3390/nu18101582)
Supplement: Supplementary file 1 [file nutrients-18-01582-s001.zip › nutrients-4016812-supplementary.pdf]

## SUPPLEMENTARY MATERIAL

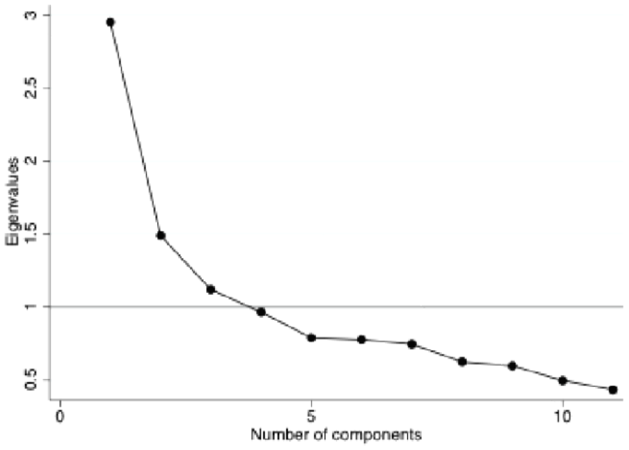


**Figure S1**: Scree plot of dietary components.

The x-axis represents the component number, and the y-axis indicates the eigenvalues of the principal components

.

**Table S1**: Food groups and items used in the principal component analysis.

| Food Group | Food items included |
|---|---|
| Vegetables | Cucumber, pumpkin, carrot, tomato, cauliflower, white radish, celtuce, bamboo shoot, broccoli, chili, potato, sweet potato, purple cabbage, cabbage, spinach, celery, rape, chrysanthemum, leaf lettuce, Chinese cabbage, garlic chives |
| Fruits | Banana, pear, apple, watermelon, jujube, mango, peach, grape, persimmon, pineapple, cherry, citrus, papaya |
| Edible fungi and algae | Kelp, mushroom, porphyria |
| Soybeans and its products | Bean sprouts, soybeans, tofu, tofu jelly, mung bean, soybean milk |
| Refined grains | Steamed bun, rice, rice-noodle |
| Nuts | Nuts |
| Red meat | Pork, beef, mutton |
| Poultry | Poultry meat |
| Fish and seafood | Freshwater fish, marine fish, shrimp and crab, seafood |
| Eggs | Eggs |
| Milk and dairy products | Pure milk, milk powder, yogurt |

**Table S2**. Characteristics of participants according to age-related macular degeneration status.

| **Variables** | **Overall, n (%)** | **Cases, n (%)** | **Controls, n (%)** | ***P*-value** |
|---|---|---|---|---|
| Age, years | 64.85±7.44 | 64.84±7.46 | 64.85±7.45 | 0.86 |
| Gender | | | | >0.99 |
| Male | 226 (45.93) | 113 (45.93) | 113 (45.93) | |
| Female | 266 (54.07) | 133 (54.07) | 133 (54.07) | |
| Educational attainment | | | | 0.04 |
| Less than college | 235 (47.76) | 129 (52.44) | 106 (43.09) | |
| College and above | 257 (52.24) | 117 (47.56) | 140 (56.91) | |
| Smoking status | | | | <0.001 |
| Yes | 95 (19.31) | 52 (21.14) | 43 (17.58) | |
| No | 397 (80.69) | 194 (78.86) | 203 (82.52) | |
| Alcohol intake | | | | <0.001 |
| Yes | 63 (12.80) | 37 (15.04) | 26 (10.57) | |
| No | 429 (87.20) | 209 (84.96) | 220 (89.43) | |
| Physical activity | | | | <0.001 |
| Low | 233 (47.36) | 128 (52.03) | 105 (42.68) | |
| Moderate | 43 (8.74) | 19 (7.72) | 24 (9.76) | |
| High | 216 (43.90) | 99 (40.24) | 117 (47.56) | |
| Sun leakage and protection | | | | <0.001 |
| Yes | 123 (25.00) | 67 (27.24) | 56 (22.76) | |
| No | 369 (75.00) | 179 (72.76) | 190 (77.24) | |
| BMI, $kg/m^2$ | 23.38±3.13 | 23.65±3.44 | 23.10±2.78 | 0.07 |
| Family history of AMD | | | | <0.001 |
| Yes | 79 (16.09) | 49 (20.00) | 30 (12.20) | |
| No | 412 (83.91) | 196 (80.00) | 216 (87.80) | |
| History of coronary heart disease | | | | <0.001 |
| Yes | 109 (22.15) | 58 (23.58) | 51 (20.73) | |
| No | 383 (77.85) | 188 (76.42) | 195 (79.27) | |
| Supplement use | | | | 0.01 |
| Yes | 208 (42.28) | 111 (45.12) | 97 (39.43) | |
| No | 284 (57.72) | 135 (54.88) | 149 (60.57) | |
| Hypertension | | | | <0.001 |
| Yes | 45 (9.10) | 24 (9.80) | 21 (8.50) | |
| No | 447 (90.90) | 222 (90.20) | 225 (91.50) | |
| Type 2 diabetes | | | | <0.001 |
| Yes | 75 (15.24) | 43 (17.58) | 32 (13.01) | |
| No | 417 (84.76) | 203 (82.52) | 214 (86.99) | |
| Total energy intake, kcal/d | 2147.40 (1635.00, 2719.38) | 2069.72 (1600.87, 2647.79) | 2209.81 (1701.09, 2818.77) | 0.05 |

AMD, age-related macular degeneration; BMI, body mass index. Continuous variables were represented as mean± standard deviation or median (*P*25, *P*75) and categorical variables were represented as frequency (%).

**Table S3:** Parallel analysis of eigenvalues and retained components in PCA

| Dietary patterns | Observed eigenvalues | Simulated 95$^{th}$ percentile eigenvalues |
|---|---|---|
| Component 1 (Prudent dietary pattern) | 2.95 | 1.30 |
| Component 2 (Egg and milk dietary pattern) | 1.49 | 1.22 |
| Component 3 (Animal foods dietary pattern) | 1.12 | 1.11 |

**Table S4:** Dietary pattern factor loading using Oblimin rotation

| Variables | Prudent dietary pattern | Egg and milk dietary pattern | Animal foods dietary pattern |
|---|---|---|---|
| Vegetables | 0.4388 | | |
| Soybeans and its products | 0.5242 | | |
| Edible fungi and algae | 0.4359 | | |
| Fruits | 0.4061 | | |
| Fish and seafood | | | 0.6161 |
| Nuts | 0.3394 | | |
| Refined grains | | 0.3237 | |
| Eggs | | 0.5340 | |
| Milk and dairy products | | 0.4959 | |
| Red meat | | | 0.3835 |
| Poultry | | | 0.6044 |
| Total initial eigenvalue % of variance explained | 20.53 | 15.54 | 14.52 |
| Cumulative % | 20.53 | 36.07 | 50.59 |

**Table S5:** Dietary pattern factor loading using Promax rotation

| Variables | Prudent dietary pattern | Egg and milk dietary pattern | Animal foods dietary pattern |
|---|---|---|---|
| Vegetables | 0.4400 | | |
| Soybeans and its products | 0.5245 | | |
| Edible fungi and algae | 0.4459 | | |
| Fruits | 0.4060 | | |
| Fish and seafood | | | 0.6201 |
| Nuts | 0.3359 | | |
| Refined grains | | 0.3225 | |
| Eggs | | 0.5342 | |
| Milk and dairy products | | 0.4965 | |
| Red meat | | | 0.3839 |
| Poultry | | | 0.6030 |
| Total initial eigenvalue % of variance explained | 20.53 | 15.54 | 14.52 |
| Cumulative % | 20.53 | 36.07 | 50.59 |

**Table S6.** Stability evaluation analysis of factor loading for the identified dietary patterns across 2,500 bootstrap replications.

| | Observed factor loading (95% CI) | Bootstrap SE | *P*-value | Percentage of bootstrap replication* |
|---|---|---|---|---|
| **Prudent dietary pattern** | | | | |
| Vegetables | 0.36 (0.31−0.41) | 0.02 | <0.001 | 99.08 |
| Fruits | 0.37 (0.33−0.41) | 0.02 | <0.001 | 99.60 |
| Edible fungi and algae | 0.34 (0.27−0.40) | 0.03 | <0.001 | 90.84 |
| Soybeans and its products | 0.38 (0.33−0.42) | 0.02 | <0.001 | 99.80 |
| Nuts | 0.29 (0.24−0.35) | 0.03 | <0.001 | 82.20 |
| **Egg and milk dietary pattern** | | | | |
| Refined grains | 0.37 (0.23−0.52) | 0.07 | <0.001 | 81.36 |
| Eggs | 0.46 (0.33−0.58) | 0.06 | <0.001 | 98.20 |
| Milk and dairy products | 0.40 (0.28−0.52) | 0.06 | <0.001 | 93.56 |
| **Animal foods dietary pattern** | | | | |
| Fish and seafood | 0.53 (-0.34−1.00) | 0.44 | 0.23 | 94.12 |
| Poultry | 0.21 (0.03−0.39) | 0.09 | 0.02 | 93.28 |
| Red meat | 0.29 (0.16−0.41) | 0.07 | <0.001 | 51.48 |

CI: confidence interval; SE: Standard Error.

* The percentage of replication refers to the percentage of 2,500 bootstrap samples in which a particular food group retained a factor loading ⩾0.30 on the corresponding principal component.

**Table S7.** Odds ratios (95% CIs) for age-related macular degeneration across tertiles of dietary pattern scores.

| Dietary Pattern | Tertiles of dietary pattern | | | $P_{trend}$ |
|---|---|---|---|---|
| | T1 | T2 | T3 | |
| **Prudent dietary pattern** | | | | |
| No of cases/controls | 100/65 | 80/84 | 66/97 | |
| Crude model | 1 | 0.68 (0.42−1.09) | 0.44 (0.26−0.74) | 0.003 |
| Multivariate model 1 | 1 | 0.69 (0.41−1.14) | 0.44 (0.26−0.77) | 0.004 |
| Multivariate model 2 | 1 | 0.52 (0.29−0.92) | 0.29 (0.14−0.59) | 0.001 |
| **Egg and milk dietary pattern** | | | | |
| No of cases/controls | 105/60 | 79/85 | 62/101 | |
| Crude Model | 1 | 0.51 (0.31−0.83) | 0.37 (0.23−0.61) | <0.001 |
| Multivariate Model 1 | 1 | 0.47 (0.28−0.78) | 0.36 (0.21−0.60) | <0.001 |
| Multivariate Model 2 | 1 | 0.50 (0.29−0.85) | 0.40 (0.23−0.67) | <0.001 |
| **Animal foods dietary pattern** | | | | |
| No of cases/controls | 79/87 | 80/82 | 87/77 | |
| Crude Model | 1 | 0.89 (0.54−1.48) | 1.09 (0.68−1.75) | 0.69 |
| Multivariate Model 1 | 1 | 0.99 (0.58−1.69) | 1.21 (0.73−2.00) | 0.46 |
| Multivariate Model 2 | 1 | 1.07 (0.62−1.86) | 1.27 (0.74−2.17) | 0.41 |

CI, confidence interval; OR, odds ratio.

Crude model: unadjusted; Multivariate model 1: adjusted for educational attainment, smoking status, alcohol consumption, physical activity, and body mass index; Multivariate model 2: additionally adjusted for supplement use, family history of AMD, history of coronary heart disease, and total energy intake

**Table S8**. Stratified analyses of dietary patterns and the likelihood of AMD.

| | OR (95%CI) | *P*-interaction |
|---|---|---|
| **Prudent dietary pattern** | | |
| Body mass index | | 0.57 |
| <24 kg/m$^2$ | 0.58 (0.37−0.90) | |
| ≥24 kg/m$^2$ | 0.77 (0.40−1.46) | |
| Physical activity | | 0.55 |
| < median | 0.73 (0.53−1.02) | |
| ≥median | 0.58 (0.23−1.43) | |
| Smoking status | | 0.68 |
| Yes | 0.16 (0.02−1.03) | |
| No | 0.70 (0.53−0.92) | |
| Alcohol consumption | | 0.06 |
| Yes | 0.70 (0.22−2.31) | |
| No | 0.61 (0.47−0.80) | |
| **Egg and milk dietary pattern** | | |
| Body mass index | | 0.37 |
| <24 kg/m$^2$ | 0.50 (0.32−0.80) | |
| ≥24 kg/m$^2$ | 0.80 (0.48−1.32) | |
| Physical activity | | 0.49 |
| < median | 0.56 (0.39−0.80) | |
| ≥ median | 0.36 (0.13−0.99) | |
| Smoking status | | 0.46 |
| Yes | 0.66 (0.26−1.67) | |
| No | 0.57 (0.43−0.76) | |
| Alcohol consumption | | 0.96 |
| Yes | 0.70 (0.30−2.02) | |
| No | 0.56 (0.42−0.73) | |

CI: confidence interval; OR, odds ratio.

**Table S9**. Sensitivity analyses of the associations between dietary components and age-related macular degeneration.

| | Tertiles of dietary pattern | | | *P* trend |
|---|---|---|---|---|
| | T1 | T2 | T3 | |
| **Prudent dietary pattern** | | | | |
| Further adjusted for the number of hours of sunlight exposure, and sun protection use | 1 | 0.43 (0.25−0.74) | 0.24 (0.11−0.49) | <0.001 |
| Further adjusted for dietary intake of lutein-zeaxanthin | 1 | 0.44 (0.26−0.76) | 0.24 (0.12−0.49) | <0.001 |
| Results within an unmatched population | 1 | 0.52 (0.32−0.85) | 0.32 (0.18−0.59) | <0.001 |
| Excluding participants with type 2 diabetes | 1 | 0.54 (0.31−0.93) | 0.35 (0.18−0.67) | 0.002 |
| Excluding participants with coronary heart disease | 1 | 0.58 (0.34−0.96) | 0.35 (0.19−0.65) | 0.001 |
| Excluding participants with hypertension | 1 | 0.73 (0.40−1.32) | 0.29 (0.14−0.60) | 0.001 |
| **Egg and milk dietary pattern** | | | | |
| Further adjusted for the number of hours of sunlight exposure, and sun protection use | 1 | 0.52 (0.31−0.87) | 0.40 (0.22−0.61) | <0.001 |
| Further adjusted for dietary intake of lutein-zeaxanthin | | 0.50 (0.30−0.84) | 0.36 (0.22−0.61) | <0.001 |
| Results within an unmatched population | 1 | 0.52 (0.33−0.82) | 0.37 (0.23−0.59) | <0.001 |
| Excluding participants with type 2 diabetes | 1 | 0.46 (0.27−0.76) | 0.35 (0.21−0.59) | <0.001 |
| Excluding participants with coronary heart disease | 1 | 0.50 (0.31−0.81) | 0.37 (0.23−0.61) | <0.001 |
| Excluding participants with hypertension | 1 | 0.45 (0.25−0.80) | 0.38 (0.21−0.67) | 0.001 |
| **Animal foods dietary pattern** | | | | |
| Further adjusted for the number of hours of sunlight exposure, and sun protection use | 1 | 1.13 (0.68−1.87) | 1.30 (0.78−2.16) | 0.31 |
| Further adjusted for dietary intake of lutein-zeaxanthin | | 1.16 (0.70−1.93) | 1.37 (0.82−2.29) | 0.23 |
| Results within an unmatched population | 1 | 1.07 (0.67−1.71) | 1.17 (0.73−1.87) | 0.51 |
| Excluding participants with type 2 diabetes | 1 | 1.14 (0.68−1.90) | 1.46 (0.87−2.45) | 0.60 |
| Excluding participants with coronary heart disease | 1 | 1.09 (0.67−1.78) | 1.34 (0.81−2.18) | 0.25 |
| Excluding participants with hypertension | 1 | 1.14 (0.64−2.04) | 1.22 (0.68−2.17) | 0.49 |

CI: confidence interval; OR, odds ratio. The model was adjusted for educational attainment, smoking status, alcohol consumption, physical activity, body mass index, supplement use, family history of AMD, history of coronary heart disease, and total energy intake.

**Table S10:** Robustness to unmeasured confounding using E-values

| **Dietary patterns** | **OR (95%CI)** | **E-value (lower bounds)** |
|---|---|---|
| Prudent dietary pattern | 0.29 (0.14−0.59) | 6.36 (2.77) |
| Egg and milk dietary pattern | 0.40 (0.23−0.67) | 4.44 (2.34) |

CI: confidence interval, OR: Odd ratio

**Table S11**: Probabilistic bias simulation results adjusting for differential misclassification

| Dietary patterns | Observed OR | Median bias-corrected ORs (95 % simulation intervals) |
|---|---|---|
| Prudent dietary pattern | 0.44 | 0.49 (0.43−0.54) |
| Egg and milk dietary pattern | 0.37 | 0.39 (0.34−0.45) |
| Animal foods dietary pattern | 1.09 | 1.21 (1.18−1.26) |

OR: Odd ratio.
